# Assessment of Potential Predictors of Aortic Stenosis Severity Using ECG-Gated Multidetector CT in Patients with Bicuspid and Tricuspid Aortic Valves Prior to TAVI

**DOI:** 10.3390/jcm15020551

**Published:** 2026-01-09

**Authors:** Piotr Machowiec, Piotr Przybylski, Elżbieta Czekajska-Chehab

**Affiliations:** Department of Radiology, Medical University of Lublin, 20-059 Lublin, Poland; dr.przybylski@gmail.com (P.P.); czekajska@gazeta.pl (E.C.-C.)

**Keywords:** bicuspid aortic valve, cardiac computed tomography, aortic valve stenosis, TAVI

## Abstract

**Background/Objectives**: The aim of this study was to evaluate the usefulness of selected predictive parameters obtainable from cardiac multidetector computed tomography for assessing the severity of aortic valve stenosis in patients scheduled for transcatheter aortic valve implantation (TAVI). **Methods**: A detailed retrospective analysis was performed on 105 patients with a bicuspid aortic valve (BAV), selected from a cohort of 1000 patients with BAV confirmed on ECG-gated CT, and on 105 patients with a tricuspid aortic valve (TAV) matched for sex and age. All patients included in both groups had significant aortic stenosis confirmed on transthoracic echocardiography. **Results**: Across the entire cohort, a trend toward higher aortic valve calcium scores was observed in patients with bicuspid compared to tricuspid aortic valves (4194.8 ± 2748.7 vs. 3335.0 ± 1618.8), although this difference did not reach statistical significance (*p* = 0.080). However, sex-stratified analysis showed higher calcium scores in males with BAV than with TAV (5596.8 ± 2936.6 vs. 4061.4 ± 1659.8, *p* = 0.002), with no significant difference observed among females (*p* > 0.05). Univariate regression analysis showed that the aortic valve calcium score was the strongest statistically significant predictor of aortic stenosis severity in both groups, with R^2^ = 0.224 for BAV and R^2^ = 0.479 for TAV. In the multiple regression model without interaction terms, the explanatory power increased to R^2^ = 0.280 for BAV and R^2^ = 0.495 for TAV. **Conclusions**: In patients scheduled for TAVI, linear regression models assess the severity of aortic stenosis more accurately than any individual predictive parameter obtainable from ECG-CT, with the aortic valve Agatston score emerging as the most reliable single CT-derived predictor of stenosis severity in both TAV and BAV subgroups.

## 1. Introduction

Aortic stenosis (AS) is the most common valvular heart defect in highly developed countries [1], affecting approximately 12.4% of individuals over 75 years of age [2]. An important element of the disease’s pathophysiology is valve calcification. The prevailing view today is that aortic valve calcification is an active process that resembles the mechanism underlying the development of atherosclerosis in blood vessels. It begins with microdamage to the valve leaflets, on which cholesterol deposits accumulate. Inflammatory factors and mechanisms associated with osteogenesis play a key role in this process [3,4,5,6].

In patients with a bicuspid aortic valve (BAV), calcification of the semilunar leaflets occurs roughly two decades earlier than in individuals with a tricuspid aortic valve. This difference is linked to the involvement of genetic factors, such as the NOTCH1 gene, in the calcification process. It is well established that the NOTCH1 gene is considered one of the factors responsible for the development of BAV [5,7,8].

Based on the 2020 ACC/AHA (American College of Cardiology/American Heart Association) guidelines, SAVR (surgical aortic valve replacement) is preferred in patients with aortic stenosis <65 years of age. In symptomatic patients aged 65–80 years, as well as in asymptomatic patients below 80 years of age whose left ventricular ejection fraction is less than 50%, SAVR is also recommended. TAVI (transcatheter aortic valve implantation) is the preferred treatment method in older patients (over 80 years of age), regardless of the degree of surgical risk, if their life expectancy is less than 10 years [9]. In line with the updated ESC/EACTS guidelines, the age cut-off favoring transcatheter aortic valve implantation (TAVI) in patients with tricuspid aortic valve stenosis has been lowered from 75 to 70 years. In addition, TAVI may be considered for the management of severe bicuspid aortic valve stenosis in patients with increased surgical risk, provided that anatomical suitability is confirmed. Notably, cardiac computed tomography–derived aortic valve calcium scoring is now regarded as equivalent to dobutamine stress echocardiography for the diagnosis of low-flow, low-gradient aortic stenosis [10]. In recent years, there has been a noticeable increase in the percentage of TAVI procedures performed more and more often in patients with bicuspid aortic valve. Despite the unusual anatomy of the valve, patients with BAV and severe aortic stenosis may experience significant benefits after TAVI, but it is necessary to select the appropriate prosthesis and use modern implantation techniques [11]. BAV is often accompanied by various coronary artery anomalies, which should be taken into account when qualifying patients for TAVI, together with an assessment of the morphological type of the bicuspid aortic valve [12].

Transthoracic echocardiography (TTE) is a well-established and widely used standard in the diagnosis of aortic stenosis. However, it is subject to inter-observer variability and may be limited by difficulties in obtaining diagnostic-quality images in certain patient groups [13]. Technical challenges in image acquisition can lead to significant underestimation or overestimation of cardiac and aortic hemodynamic parameters, directly influencing diagnosis, prognosis, and treatment strategies. Echocardiographic assessment of aortic stenosis severity typically relies on measurements of peak velocity, maximum and mean pressure gradients (MPG), and aortic valve area (AVA), which ideally should be mutually consistent. In 20–30% of patients, these parameters are discordant (most commonly when AVA < 1 cm^2^ and MPG < 40 mmHg) [14]. When the degree of stenosis cannot be reliably assessed by echocardiography during TAVI qualification, evaluation of valve calcification using the computed tomography–derived Calcium Score is recommended [15]. The most commonly cited cut-off values for defining severe aortic stenosis are 2000 for men and 1300 for women [16].

In preoperative planning prior to TAVI, cardiac computed tomography plays an important role in assessing the anatomy of the aortic annulus and left ventricular outflow tract, the height of the coronary ostia, the presence of valve calcifications, and the selection of the appropriate prosthesis size. In addition, computed tomography is helpful in selecting vascular access and predicting potential perioperative complications [17,18]. Currently, valvular calcification is most often measured based on native scanning. The assessment of calcification after contrast agent administration is difficult due to the lack of standardized cut-off points that allow differentiation between calcification and contrast-filled aortic lumen. However, if repeatable and reliable results could be obtained using standardized cut-off points, this could prove to be a useful tool. It would eliminate the need for non-contrast CT to assess valvular calcification, thereby reducing examination time and unnecessary radiation exposure [19,20].

Assessment of the aortic valve calcification score (AVCS) is a complementary method for evaluating the severity of aortic stenosis relative to echocardiography, with the advantage of being independent of hemodynamic conditions and cardiac loading conditions [21]. AVCS is particularly useful in assessing the severity of aortic stenosis in patients with low-flow, low-gradient AS (LF-LG AS), in whom traditional methods may be insufficient. In such cases, a high AVCS may indicate truly severe stenosis, even when echocardiography suggests a less severe degree of AS [20,22]. It has been demonstrated that the degree of aortic valve calcification correlates positively with stenosis severity assessed by echocardiography. Moreover, recent meta-analyses confirm that AVCS measured using multidetector computed tomography (MDCT) allows for the diagnosis of severe aortic stenosis with a sensitivity of 82% and a specificity of 78% [23,24]. The usefulness of the calcification score may be limited in patients with a small aortic annulus area and in young people, in whom stenosis is more often associated with fibrous lesions [25].

There is extensive literature on the echocardiographic assessment of aortic stenosis, both in patients with tricuspid and bicuspid aortic valves [26,27,28,29,30]. In addition, the utility of the aortic valve calcification score obtained from computed tomography in evaluating the severity of aortic stenosis has been comprehensively described [16,19,31,32,33]. Therefore, the aim of this study is to analyze the usefulness of additional auxiliary predictive parameters obtainable from multislice cardiac computed tomography for assessing the severity of aortic valve stenosis, with particular consideration of the aortic valve type.

## 2. Materials and Methods

### 2.1. Study Design

**Figure 1:**
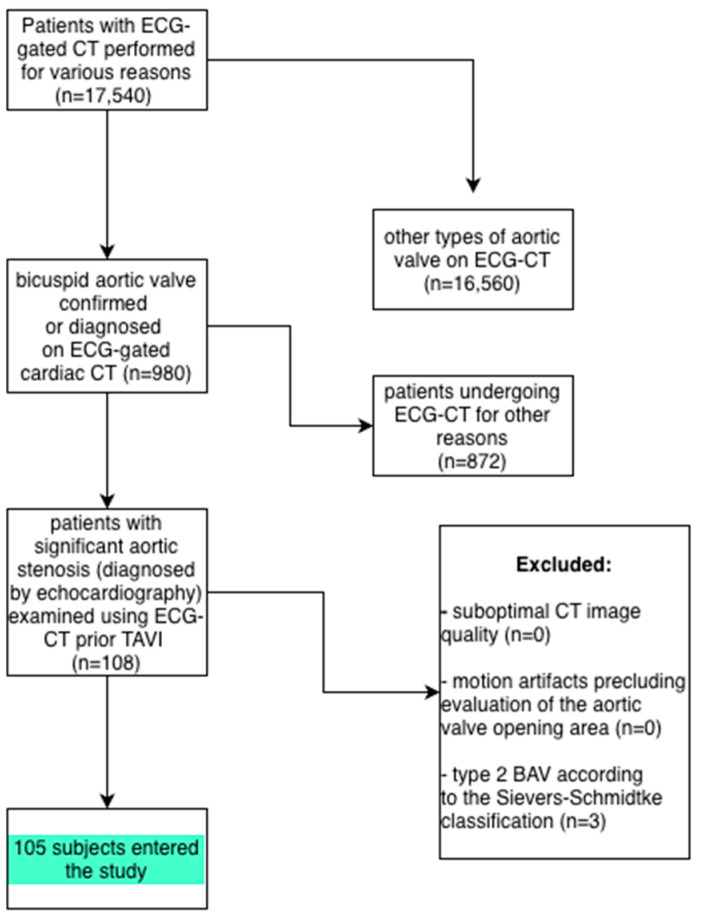
Study flowchart with inclusion and exclusion criteria.

A retrospective analysis was performed in a cohort of 210 patients with significant aortic stenosis diagnosed by echocardiography, including 105 individuals with a bicuspid aortic valve (BAV) and 105 with a tricuspid aortic valve (TAV), all of whom underwent multislice ECG-gated cardiac CT prior to planned TAVI. The BAV group (n = 105) was selected from approximately 1000 patients in whom BAV morphology had been identified by ECG-CT. The size of the TAV group (n = 105) was determined to match the BAV group in terms of age and sex distribution. At the initial stage, 3 patients with Sievers–Schmidtke type 2 bicuspid aortic valves (the so-called unicuspid variant) were excluded; no exclusions were related to suboptimal CT image quality or motion artifacts (Figure 1). For the analysis of relationships between the CT-derived aortic valve area and both the transvalvular aortic pressure gradients and echocardiographic AVA, only echocardiographic studies performed in our center were included. This approach aimed to minimize inter-operator variability and ensure consistent, reliable measurements.

Echocardiographic studies were included in the analysis only if at least one of the following parameters was available: maximum transvalvular pressure gradient, mean transvalvular pressure gradient, or aortic valve area. When identifying potential predictive parameters of aortic stenosis severity, we considered only those measurable in ECG-gated cardiac CT. These included: aortic valve calcium score, calcium score indexed to the aortic annulus area, aortic annulus area, left atrial volume, left ventricular ejection fraction, aortic contractility, aortic strain, and—within the BAV subgroup—the morphological type of the bicuspid aortic valve.

### 2.2. BAV Classification

In our study, we used two classifications of patients with bicuspid aortic valve (BAV): the modified Sievers–Schmidtke classification and the Jilaihawi classification. The latter was subsequently used in the linear regression analysis. Aortic valve morphology was assessed in all phases of the cardiac cycle; however, the phase of full opening (15%) and the phase of complete closure (75%) were typically selected for detailed evaluation. According to the Sievers–Schmidtke classification [34], we distinguished type 0, characterized by the presence of two cusps, two zones of parallel apposition, and two commissures, and type 1, defined by the presence of a single raphe located on one of the cusps. Type 2 was excluded from the analysis, as some researchers consider it not to represent a true bicuspid valve but rather an unicuspid aortic variant. Additionally, BAV types were further subdivided based on cusp orientation in type 0 (anterior–posterior and lateral) and based on raphe location in type 1, distinguishing the following subtypes: R–L (right–left), N–L (non-coronary–left), and N–R (non-coronary–right).

The classification proposed by Jilaihawi [35] is a simplified, non-numerical classification used mainly in patients referred for TAVR, based on the diversity of valve leaflet morphology, and distinguishes three morphological types of BAV:

- Tricommissural—often referred to as the “functional/acquired” subtype, one commissure is completely fused between two cusps, while fusion is not seen in the basal third of the sinus;
- Bicommissural raphe type—two cusps are fused by a fibrous or calcified ridge that does not extend to the level of the commissure;
- Bicommissural non-raphe type—two cusps are completely fused from their basal origin, with no visible seam.

### 2.3. Computed Tomography Protocol

The examinations were performed at the Department of Radiology and Nuclear Medicine of the University Clinical Hospital No. 4 in Lublin using a 256-slice Revolution CT scanner (General Electric Healthcare, Milwaukee, WI, USA). They were performed by means of native scanning (calcium scoring) with ECG gating to assess valvular calcification and after intravenous administration of a contrast agent. In most of the patients, scan extent covered the area from the aortic arch to the femoral arteries. However, due to the increase in the number of potential vascular access sites, in some cases the scan extent was individually extended. The technical parameters of computed tomography angiography for scanners were as follows: collimation 256 × 0.625 mm, tube rotation time 0.28 s. In most subjects, a tube voltage of 120 kV was used, but in some patients, due to weight and age, the tube voltage was adjusted individually and was either 80, 100, or 140 kV. The amount of contrast agent was 1 mL/kg (70–140 mL). The iodine contrasts used were either Iomeron 400, 400 mg I/mL (Bracco Imaging Deutschland, Konstanz, Germany) or Ultravist 370, 370 mg I/mL (Bayer Pharma AG, Berlin, Germany) administered at a flow rate of 4.5–6 mL/s. The scan delay was determined using the SmartPrep technique in the ascending aorta. In some clinical indications, it was individually decided to perform the second phase of scanning after 60 s, e.g., when a thrombus was suspected in the left atrial appendage. Subsequently, images were reconstructed in 10 series with a 10% R-R interval starting from phase 5% (5–95%). After obtaining the reconstructed series, all data were sent to one of the dedicated diagnostic consoles (Advantage Window 4.6 or 4.7 from GE) with software for evaluating cardiac tomography examinations (CardIQ, https://www.gehealthcare.com/products/advanced-visualization/all-applications/cardiq-suite; accessed date 20 June 2025). Studies were performed in axial planes, multiplanar reconstructions (MPRs), maximum-intensity projections (MIPs), and volume reconstructions.

### 2.4. Mathematical Formulas and Measurements

The study used several mathematical equations presented below:

$${\mathrm{Ao}}_{\mathrm{strain}}\;=\;\frac{Ao\;systolic\;diameter-Ao\;diastolic\;diameter}{Ao\;diastolic\;diameter}\times 100\%$$

$${\mathrm{Ao}}_{\mathrm{contractility}}=\frac{Ao\;systolic\;area-Ao\;diastolic\;area}{Ao\;diastolic\;area}\times 100\%$$

$$X_{c}=X_{i}-\bar{x}$$

where:

X_c_—centered variable; X_i_—variable value for observation i; $\bar{x}$—arithmetic mean of a variable X.

The aortic valve area (AVA) was assessed at the phase of full valve opening, typically at 15–25% of the R–R interval. The cross-sectional area and diameter of the ascending aorta were measured at its widest point in both the end-systolic and end-diastolic phases. Based on these values, aortic strain and aortic contractility were calculated as described above. Due to conformational changes in the aortic annulus throughout the cardiac cycle, the annulus area was measured in the systolic phase. The left atrial volume was assessed at maximum filling, usually at 35–45% of the R–R interval. In addition to calculating the aortic valve calcium score (AVCS) using the SmartScore 4.0 tool, an indexed calcification score—defined as AVCS divided by the aortic annulus area (Ca/AVAA)—was also analyzed.

### 2.5. Statistical Analysis

Statistical analysis was performed using Statistica 13.1 software (Statsoft, Palo Alto, CA, USA). Age in individual patient groups is presented as a range of values, median, and interquartile range. Other quantitative variables are expressed as mean and standard deviation, while qualitative variables are presented as numbers and corresponding percentages. Qualitative data were compared using the χ^2^ test, while Student’s *t*-test or Mann–Whitney test were used for comparative analysis of quantitative data. The choice of statistical tests in the comparative analyses of quantitative values depended on their distribution, which was assessed using the Shapiro–Wilk test for normality. For variables not meeting the assumptions of normality or non-parametric variables, the Mann–Whitney test was used.

The predictive values of several potential parameters obtained in CT were assessed, initially using univariate linear regression analysis. The dependent variable in the analysis was the aortic valve area (AVA, cm^2^). The following independent variables were included in the analysis: Agatston score (aortic valve calcium), indexed Agatston score, aortic strain, aortic contractility, aortic annulus area, left atrial volume, left ventricular ejection fraction, and valve subtype according to the Jilaihawi classification in the BAV group. Each parameter was assigned a specific R^2^ determination coefficient value, and *p* < 0.05 was considered statistically significant.

In the next stage, classical multiple linear regression was performed using all main variables (full model). Based on this initial set of predictors, backward stepwise regression was applied with the Akaike Information Criterion (AIC), sequentially removing the least significant variables until an optimal model containing only statistically significant predictors (*p* < 0.05) was obtained. The Akaike Information Criterion (AIC) was selected as the primary criterion for model selection rather than relying solely on *p*-values. While *p*-value–based approaches assess individual predictors separately, AIC evaluates overall model performance by balancing goodness-of-fit and model complexity. This strategy reduces the risk of overfitting and limits the exclusion of predictors whose effects may not be apparent in univariate analyses but emerge after multivariable adjustment. In addition, *p*-values are influenced by sample size and collinearity among predictors, which may result in unstable variable selection. By contrast, AIC allows for more consistent comparison of competing models with different predictor combinations. Therefore, some parameters were retained in the final models despite marginal or non-significant univariate associations when their inclusion led to a lower AIC and improved model fit. The final model was then evaluated for compliance with classical linear regression assumptions, including linearity, absence of autocorrelation, homoscedasticity, normality of residuals, and absence of problematic collinearity. To limit the impact of multicollinearity, variance inflation factors (VIF) were calculated, with a threshold of VIF > 5 indicating significant multicollinearity.

In addition, models were also built taking potential interactions between variables into account. In addition to the main variables, statistically significant interactions were included in the initial models subjected to backward stepwise regression. In cases where some interactions had higher VIF values, the main variables were centered in order to reduce the correlation of the interaction with its components. For the final linear regression models, residuals vs. fitted graphs, histograms of residuals, and Q-Q plots were created, as well as linear regression equations, expressed as:

Y = a + b_1_X_1_ + b_2_X_2_ + … b_n_X_n_ + ε

where:

Y—dependent variable;

a—intercept;

X_1_, X_2_, …, X_n_—various independent variables;

b_1_, b_2_, …, b_n_—regression coefficients that show the effect of each variable on Y.

Variable aortic strain and aortic contractility were substituted into the equation in fractional form, not as a percentage.

## 3. Results

### 3.1. Characteristics of Study Group

The study group consisted of 210 patients—105 patients with a bicuspid aortic valve and 105 patients with a tricuspid aortic valve, matched for gender and age. The age range was 59 to 90 years, with a median of 77 and IQR 72–80. In each group, females accounted for 54.3% (57 cases), which was determined by the number of females with BAV. In the male group, the age range was 59–90, Me 76, IQR 73–76, while for females it was 62–90, Me 77, IQR 72–81. The characteristics of the study group are presented in Table 1.

The group of patients with bicuspid aortic valve was divided based on the modified Sivers–Schmidtke classification and the classification proposed by Jilaihawi et al., as presented in Table 2 and Table 3. Type 1 RL predominated in the Sivers–Schmidtke classification—68.6% of patients, while in the second classification, the tricommissural and bicommissural raphe-type prevailed—each occurring in 44.8% of cases.

In the first stage of the study, we analyzed the relationship between the aortic valve area calculated in cardiac computed tomography and the maximum aortic gradient, mean aortic gradient, and aortic valve area in echocardiography, both in the BAV and TAV groups (Table 4).

The mean aortic valve area measured in computed tomography was 153.2 ± 41.2 mm^2^ in the group of patients with TAV and 167.5 ± 53.3 mm^2^ in the group with BAV. In the TAV group, AVA_CT_ correlated significantly with the maximum aortic gradient (strongest, R = −0.50), mean aortic gradient, and valve area obtained in echocardiography. A statistically significant relationship was found between AVA_CT_ and the maximum aortic gradient (strongest, R = −0.36) and the mean aortic gradient. However, no statistically significant correlation was observed between AVA_CT_ and AVA_echo_ in the BAV group (n = 30), *p* = 0.180.

**Figure 2:**
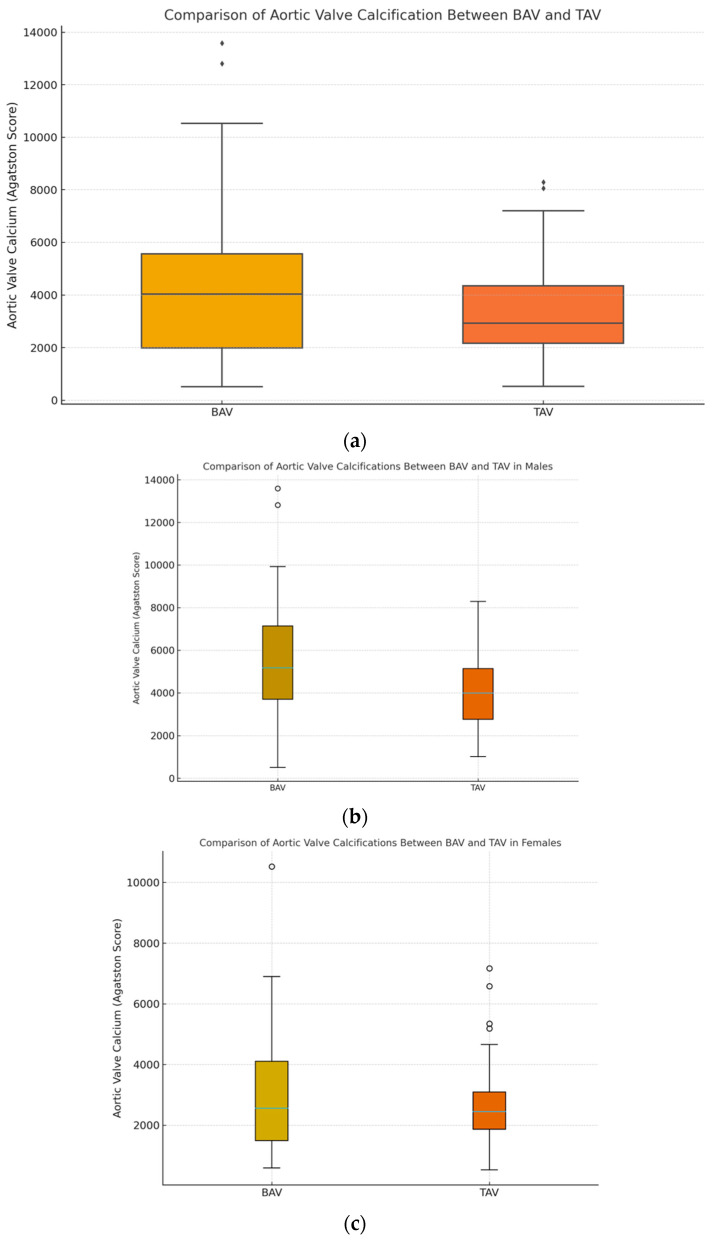
(**a**) Comparison of valve calcifications in the BAV and TAV groups—box-and-whisker plot, N = 210, *p* = 0.080. (**b**) Comparison of valve calcifications in the BAV and TAV groups in males—box-and-whisker plot, n = 98, *p* = 0.002. (**c**) Comparison of valve calcifications in the BAV and TAV groups in females—box-whisker plot, n = 112, *p* = 0.789.

An analysis of valvular calcification was performed in a group of patients with bicuspid and tricuspid aortic valves. In the entire analyzed group, a tendency toward higher aortic valve calcium scores was observed for bicuspid aortic valves compared to tricuspid valves (4194.8 ± 2748.7 vs. 3335.0 ± 1618.8), but these differences did not meet the strict criteria for significance (*p* = 0.080) (Figure 2a). Additionally, an analysis stratified by gender revealed significant differences in the aortic valve calcium score among males (BAV 5596.8 ± 2936.6 vs. TAV 4061.4 ± 1659.8, *p* = 0.002) (Figure 2b). In contrast, no significant differences in the AVCS were observed in females (BAV 3014.09 ± 1920.78 vs. TAV 2699.43 ± 1292.11, *p* = 0.789) (Figure 2c).

The next step focused on searching for alternative or auxiliary predictive parameters, obtainable in CT, for assessing the severity of aortic stenosis. These parameters are presented in detail in Table 5. The characteristics of valve (BAV) subtypes are presented in Table 3.

As a result of the simple linear regression analysis, it was found that statistically significant predictors of aortic stenosis severity in the group of patients with BAV included the Agatston score (β = −0.009, R^2^ = 0.224) and the Agatston score indexed to the aortic annulus area (Ca/AVAA) (β = −0.064, R^2^ = 0.218). In the TAV group, statistically significant results were obtained for the Agatston score (β = −0.018, R^2^ = 0.479), the Agatston score indexed to the aortic annulus area (β = −0.068, R^2^ = 0.389), the aortic annulus area (β = −0.058, R^2^ = 0.041), and the left atrial volume (β = −0.253, R^2^ = 0.075) (Table 6).

Next, a multivariate analysis was performed in both the BAV and TAV groups, starting with a model that included all variables and subsequently removing those that were not statistically significant. In constructing the final models, the Agatston score was used instead of the Ca/AVAA index, as it demonstrated better fit in both the univariate model and the preliminary multivariate models.

### 3.2. TAV Regression Models

**Figure 3:**
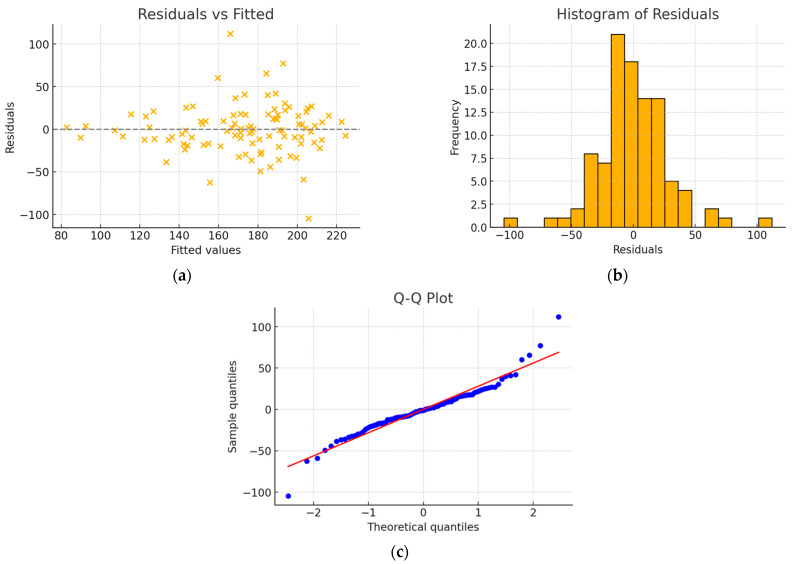
(**a**) Residuals vs. fitted plot; (**b**) histogram of residuals; (**c**) Q-Q plot—model without interaction. The residuals are scattered around zero with no discernible pattern. The histogram of residuals shows a distribution close to normal, with slight asymmetry. The Q-Q plot indicates that the residuals are well aligned with a normal distribution, with minor deviations at the tails.

In the group of patients with a tricuspid aortic valve, the final model constructed using backward regression consisted of four statistically significant predictive variables (Table 7) and had an R^2^ value of 0.534. No issues with residual normality or homoscedasticity were observed (Figure 3).

The model equation is presented below:

*AVA* = 229.650 − 0.020 × Agatston score + 0.097 × Aortic annulus area − 165.260 × Aortic contractility − 0.170 × Left atrial volume

Taking into account the higher VIF values for aortic annulus area (VIF = 28.0) and left atrial volume (VIF = 17.1), an analysis of model fit and stability was performed after removing one of these variables. It was found that after excluding the aortic annulus area, the R^2^ value decreased only slightly to 0.500, while none of the remaining model components reached a VIF > 5, which improved the model’s stability and interpretability.

**Figure 4:**
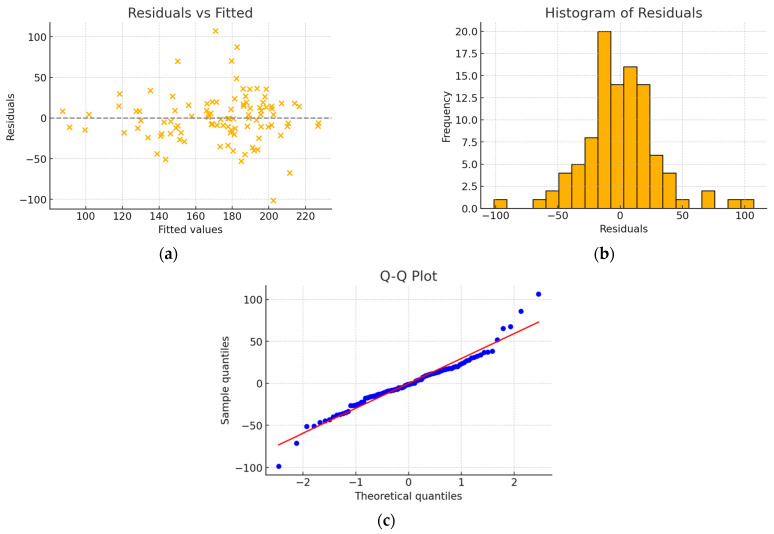
(**a**) Residuals vs. fitted plots; (**b**) histogram of residuals; (**c**) Q-Q plot—model without interaction, after excluding the left atrial volume and aortic annulus area variables. The residuals are randomly distributed, with no discernible pattern. The histogram of residuals shows a distribution close to normal. The Q-Q plot reveals slight deviations from linearity, mainly at the tails.

Due to the moderate correlation between left atrial volume and aortic annulus area, after rejecting aortic annulus area, the left atrial volume variable became statistically insignificant (*p* > 0.05) in the proposed model. A 2-variable model was created, as shown below (Table 8), characterized by R^2^ = 0.495. No problems with normality of residuals or homoscedasticity were observed (Figure 4).

Ultimately, the final model can be expressed by the following equation:

*AVA* = 229.650 − 0.018 × Agatston score − 142.205 × Aortic contractility

**Figure 5:**
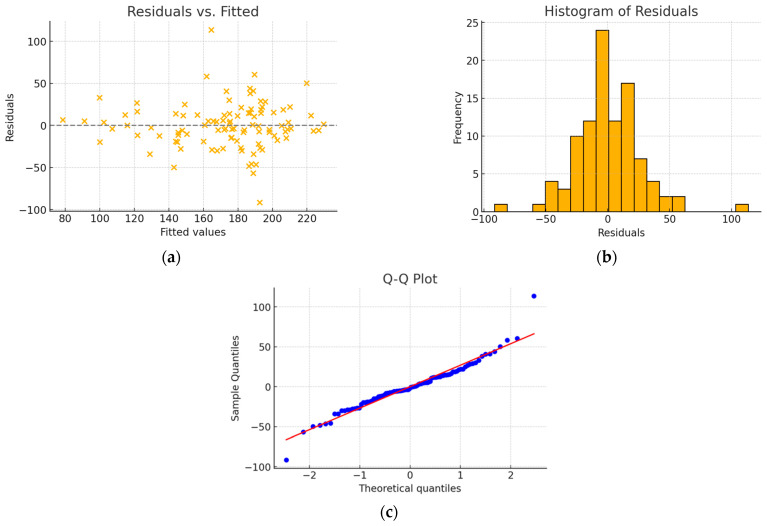
(**a**) Residuals vs. fitted plot; (**b**) histogram of residuals; (**c**) Q-Q plot—model with interactions. The residuals are randomly distributed, with no discernible patterns. The histogram of residuals shows a distribution close to normal, with slight asymmetry. The Q-Q plot reveals minor deviations from linearity, primarily at the tails.

In the next step, potential interactions between independent variables were analyzed. Statistically significant interactions included aortic annulus area × ejection fraction (*p* = 0.04) and left atrial volume × ejection fraction (*p* = 0.03). These interactions were introduced into the initial model, which was subjected to backward stepwise regression until a model consisting exclusively of statistically significant variables was obtained. The variables forming the final model were centered, achieving satisfactory VIF values and improving the interpretability of the model. After centering, ejection fraction was included in the model, as its *p*-value was close to the significance level. The model fit level was R^2^ = 0.576 (Table 9). No problems with normality of residuals or homoscedasticity were found (Figure 5).

The expanded model was constructed based on centered variables (i.e., the mean was subtracted from each variable value). The final model equation is as follows:

*AVA* = 229.650 − 0.020 × (Agatston score − 3335) + 0.089 × (Aortic annulus area − 514) − 212.983 × (Aortic contractility − 0.067) − 0.170 × (Left atrial volume − 167) − 0.433 × (Ejection fraction − 64) − 0.006 × [(Aortic annulus area − 514) × (Ejection fraction − 64)] + 0.011 × [(Left atrial volume − 167) × (Ejection fraction − 64)]

### 3.3. BAV Regression Models

**Figure 6:**
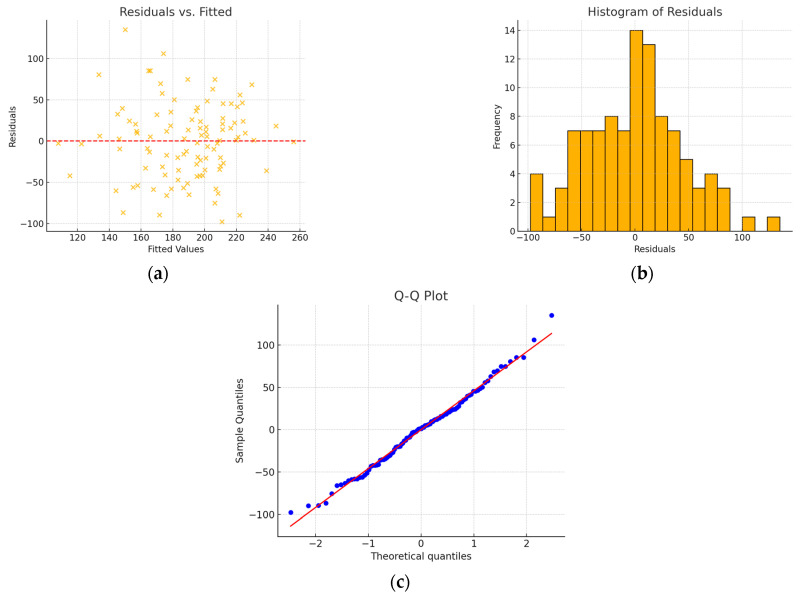
(**a**) Residuals vs. fitted chart; (**b**) histogram of residuals; (**c**) Q-Q plot—model without interaction. The absence of a clear pattern (e.g., funnel shape or curvature) indicates that the assumptions of linearity and homoscedasticity are met. The distribution of residuals is close to normal. The points on the Q-Q plot are arranged along a straight line, confirming the normality of the residual distribution, with minimal deviations at the tails.

In the group of patients with bicuspid aortic valve, the multiple analysis model, without taking into account the interaction between variables, was characterized by a value of R^2^ = 0.280 (Table 10). The statistically significant parameters forming the final regression model include the Agatston score and aortic strain, as well as aortic annulus area, which is a variable close to statistical significance in the presented model, but improves the fit of the model itself. No problems were observed with the normality of residuals, homoscedasticity, or significant collinearity of the variables forming the model (Figure 6).

The final equation of the model is as follows:

*AVA* = 165.963 − 0.011 × Agatston score + 795.900 × Aortic strain + 0.077 × Aortic annulus area

**Figure 7:**
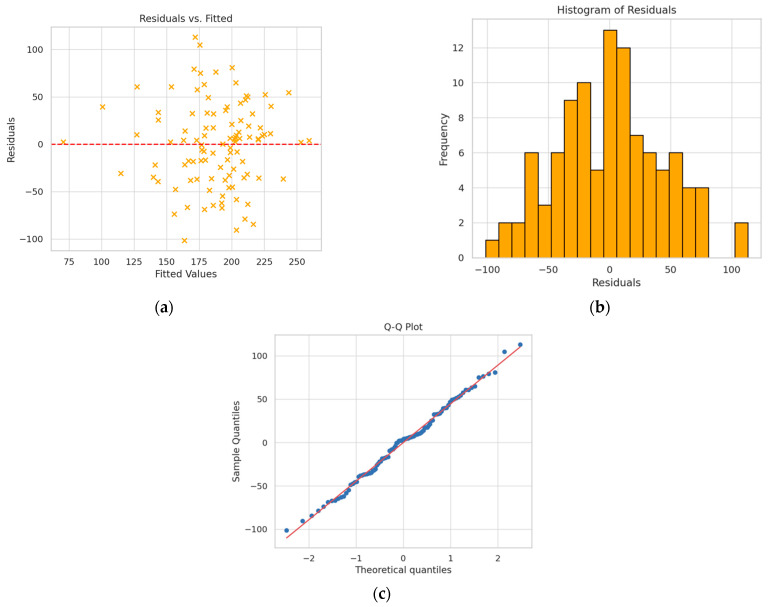
(**a**) Residuals vs. fitted plot; (**b**) histogram of residuals; (**c**) Q-Q plot—model with interaction. The residuals are randomly scattered around zero, with no discernible patterns or signs of heteroscedasticity. The residual distribution is approximately normal, with a slight right skew. The points on the Q-Q plot lie along a straight line, confirming the normality of the residuals, with only minimal deviations at the extremes.

In the next step, a model accounting for interactions between variables was created to improve model fit (Table 11). A statistically significant interaction was found between the Agatston score and tricommissural BAV type. The final model, obtained using stepwise regression, had an R^2^ value of 0.324 and included only the variables/interactions presented in the table below. The categorical variable “tricommissural BAV” was included because, although its main effect was not significant (*p* = 0.11), its interaction with the Agatston score was significant (in accordance with the hierarchical principle). All variance inflation factor (VIF) values for the variables were below 5, indicating no significant multicollinearity. No significant issues with homoscedasticity or residual normality were observed (Figure 7).

The equation of the model taking interactions into account is presented below:

*AVA* = 146.849 − 0.008 × Agatston score + 874.633 × Aortic strain + 0.088 × Aortic annulus area + 26.281 × tricommissural BAV − 0.008 × (Agatston score × tricommissural BAV)

**Figure 8:**
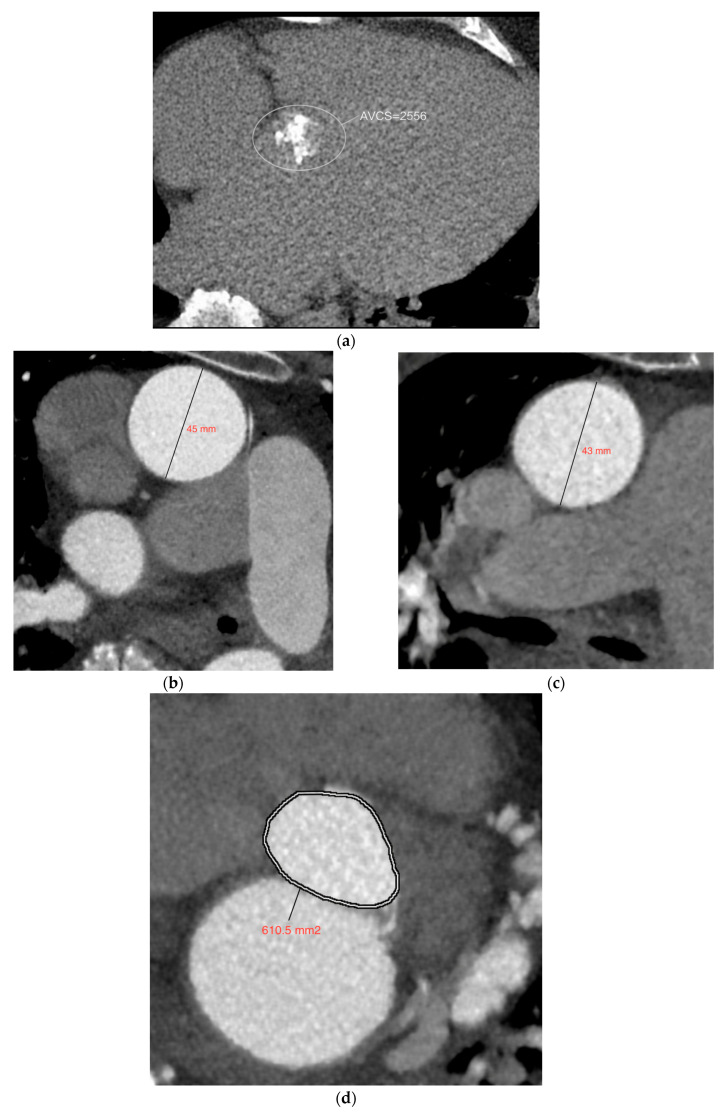
Measurements of particular predictive parameters of the aortic valve area in a patient with BAV: (**a**) aortic valve calcium score (AVCS); (**b**,**c**) diameter of the ascending aorta in systole (left side) and diastole (right side); (**d**) aortic annulus area.

Below, the measurements of the individual predictive parameters and the method for calculating *AVA*_CT_ are presented, using the example of a patient with BAV and a model composed solely of the main variables (Figure 8).

The results obtained were substituted into the following equation:

*AVA* = 165.963 − 0.011 × Agatston score + 795.900 × Aortic strain + 0.077 × Aortic annulus area

*AVA* = 165.963 − 0.011 × 2556 + 795.900 × 0.047 + 0.077 × 610.5

*AVA* ≈ 222

**Figure 9:**
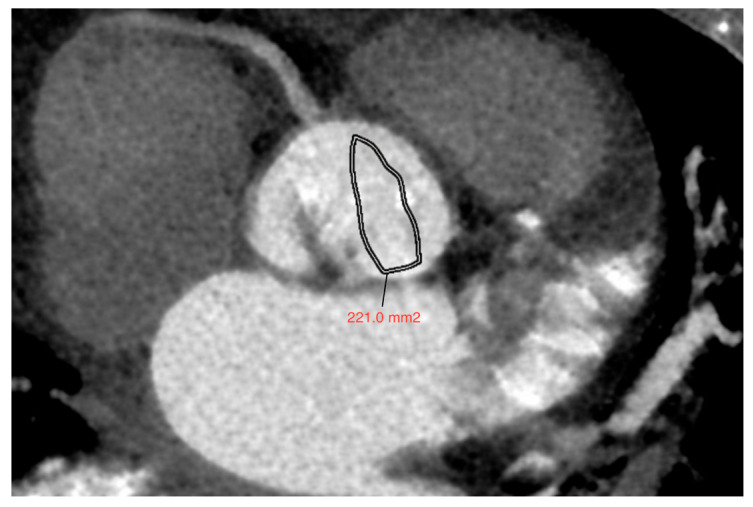
A 67-year-old female patient with a bicuspid aortic valve. The aortic valve area (AVA_CT_) calculated in computed tomography—221 mm^2^ was similar to the area obtained using the proposed regression model.

The result obtained was similar to the aortic valve area measured by computed tomography (AVA_CT_) (Figure 9).

## 4. Discussion

In recent years, the number of transcatheter aortic valve implantation (TAVI) procedures performed in patients with aortic stenosis, both with tricuspid and bicuspid aortic valves, has increased significantly. Aortic valve stenosis in patients with bicuspid aortic valve (BAV) is associated with a higher degree of valvular calcification, primarily due to hormonal, genetic, cellular, inflammatory, and possibly hemodynamic factors [11]. As demonstrated by Leopold et al., it leads to a more frequent need for pre-dilatation of the valve with a balloon during TAVI in this patient group [36]. Additionally, BAV is often associated with various coronary artery anomalies, which should be considered during patient selection for TAVI. It has been shown that patients with BAV without the presence of a raphe and with an orifice that is vertically oriented may be at higher risk of coronary ostial occlusion during the procedure due to the short distance between the ostia, as demonstrated by Nikas et al. [11]. Patients with BAV may also present with mixed valvular disease (stenosis with regurgitation). Halim et al. emphasized that although such patients may be candidates for TAVI, available data on outcomes in this subgroup are limited [37].

Similar to other researchers [11], in our study we found a tendency for higher calcium scores in the BAV group compared to the TAV group, as assessed by CT prior to planned TAVI (4194.8 ± 2747.7 vs. 3335.0 ± 1618.8). However, these differences did not meet strict significance criteria (*p* = 0.08), which may have been influenced by the relatively large proportion of women in the study group. In our study, we found statistically significant differences in the valve calcium score between males in the BAV and TAV groups (BAV 5596.8 ± 2936.6 vs. TAV 4061.4 ± 1659.8, *p* = 0.002). However, no significant differences in the valve calcium score were found in the female groups, which may be related to larger deposits of fibrous elements on the aortic valve leaflets in the female group as reported by Iribarren et al. [38]. Van Rosendael et al. found that calcifications within BAV leaflets are often asymmetrical and most commonly affect the left leaflet [39]. According to Kong et al., the amount and distribution of valvular calcifications can pose a challenge for transcatheter aortic valve implantation, but new-generation devices are able to overcome the anatomical and calcification-related difficulties associated with BAV [12].

According to recent studies, the aortic valve calcium score indexed to the aortic annular area (AVCd—aortic valve calcium density) appears to be an equivalent or even superior parameter for assessing the severity of aortic stenosis compared with the classic calcium score, particularly in patients with inconclusive echocardiographic findings or low-flow aortic stenosis [31]. As reported by Gomes et al., the indexed calcification score enhances the classification of aortic stenosis severity, especially within the so-called “gray zone,” defined as calcium score values of 800–1200 AU in women and 1600–2000 AU in men [40]. In addition, Powers et al. proposed AVCd cut-off values for severe aortic stenosis of 300 AU/cm^2^ for women and 500 AU/cm^2^ for men [31]. In our study, conducted in a group of patients with aortic stenosis evaluated prior to TAVI, the calcium score indexed to the annular area demonstrated a slightly lower coefficient of determination (R^2^) in univariate regression compared with the non-indexed calcium score. This indicates that AVCd explained less of the variance in the dependent variable (R^2^: 0.224 vs. 0.218 in the BAV group and 0.479 vs. 0.389 in the TAV group).

Overall, current guidelines provide a consistent definition of severe aortic stenosis. In addition to the classical high-gradient form, characterized by a peak aortic jet velocity ≥4 m/s, severe AS may also be present in cases with lower peak velocity or mean transvalvular gradient (<4 m/s or <40 mmHg). In patients with low-gradient AS and reduced left ventricular ejection fraction (LVEF ≤ 50%), referred to as classical low-flow low-gradient AS, further evaluation with dobutamine stress echocardiography and/or non-contrast computed tomography (CT) for aortic valve calcium scoring is recommended. CT calcium scoring is also advised in patients with preserved LVEF (>50%) when the aortic valve area is <1.0 cm^2^ and the stroke volume index is reduced (<35 mL/m^2^), consistent with paradoxical low-flow low-gradient AS. Both American and European guidelines emphasize that assessment of paradoxical low-flow low-gradient AS severity should rely on an integrative evaluation incorporating echocardiographic findings, CT-derived aortic valve calcium scores, and clinical parameters [41]. For the purposes of the present study, the same definition of severe aortic stenosis was applied.

In the available literature, apart from the aortic valve calcium score, the predictive values of other parameters in the context of aortic stenosis were analyzed. Raghunathan et al. compared echocardiographic circumferential strain of the ascending aorta (CSAA) in patients with normal aortic valves and severe aortic stenosis prior to TAVR. A significant difference in CSAA values was observed between patients with normal ascending aorta and those with severe aortic valve stenosis—10% vs. 4%, respectively (*p* < 0.01) [42]. Furthermore, according to Notaney et al., aortic stiffness is an important factor contributing to increased afterload in aortic stenosis, leading to adverse cardiac remodeling [43]. In our univariate regression analysis, both aortic strain and aortic contractility proved to be statistically insignificant parameters in the assessment of aortic stenosis severity in the BAV and TAV groups. Nevertheless, aortic contractility proved to be a statistically significant variable in the multiple regression model in the TAV group, while aortic strain proved to be statistically significant in the BAV group. It was probably due to the masking effect (suppressor variable) and/or a reduction in the standard error of estimation as a result of adding new variables to the model. 

Another potential parameter of aortic stenosis severity may be left ventricular ejection fraction. As aortic valve stenosis progresses, the left ventricle hypertrophies to compensate for the excessive afterload on the left ventricle and normalize cardiac wall stress [44,45]. As noted by Spilias et al., approximately one-third of patients with severe AS exhibit reduced left ventricular systolic function [46]. Saito et al. evaluated LVEF trajectories in patients with newly diagnosed severe AS who had undergone at least one echocardiographic examination before diagnosis.

It turned out that the deterioration of the left ventricular ejection fraction began even before severe aortic stenosis was noted in patients with LVEF < 50% and intensified after the aortic valve area decreased to <1.2 cm^2^. In contrast, mean LVEF remained above 60% in more than 60% of individuals whose LVEF was ≥50% at the time of initial diagnosis [47]. As reported by Dahl et al., although the prognostic value of LVEF after surgical aortic valve replacement is well established [48], considerably less is known about the predictive value of this parameter for grading AS severity. As an isolated independent variable, left ventricular ejection fraction did not show predictive value in assessing the severity of aortic stenosis in the BAV and TAV groups, which partially confirmed the fact that a significant proportion of patients with aortic stenosis (including severe stenosis) have preserved ejection fraction. However, in the TAV group, within a model incorporating interaction terms, statistically significant interactions were identified between EF and aortic annulus area (*p* = 0.04) and between EF and LAV (*p* = 0.03). These relationships contributed to a final model fit of R^2^ = 0.576. Variable centering was applied to reduce multicollinearity to acceptable levels. As a result, the EF variable became close to statistically significant (*p* = 0.06) (before centering, it was strongly associated with interactions) and was additionally included in the model, providing independent information and improving the model fit. Other researchers have found a link between the severity of aortic stenosis and the geometric and functional parameters of the left atrium. Saeed et al. indicated in their study that in the course of aortic valve stenosis, chronic pressure overload of the left ventricle results in its hypertrophy, relaxation disorders, increased wall stiffness, fibrosis processes and secondary enlargement of the left atrium (LA). LA enlargement may reflect chronic diastolic dysfunction and be an indicator of a more advanced stage of the disease in patients with aortic stenosis [49]. In a study by Sanchez-Lezama et al., the end-systolic volume of the left atrium assessed by echocardiography increased by 32% in the group of patients with moderate aortic stenosis (*p* < 0.05) and by 33% in those with severe aortic stenosis. However, no significant difference in LA size was observed between the moderate and severe groups, and the study population was relatively small [50]. Cionca et al. found that patients with severe aortic valve stenosis experience significant structural and functional changes in the left atrium, including impaired function in individual phases of the cardiac cycle and reduced deformability, reflecting advanced atrial remodelling. This was the first study to evaluate left atrial parameters in patients with aortic stenosis using cardiac magnetic resonance imaging, including the left atrial sphericity index (LASI), left ventricular mass (LVM) and left atrial volume (LAV) [51]. In our CT-based analysis, we assessed only LA volume. It proved to be an independent, statistically significant predictor of AS severity in the TAV group (*p* = 0.005), although it explained only a small proportion of the variance in the dependent variable (R^2^ = 0.075). Nevertheless, LAV served as an important supplementary predictor within the multivariable regression models in this group.

The aortic annulus, a tissue structure surrounding the aortic valve, plays a fundamental role both in the pathophysiology of aortic stenosis and in the planning of TAVI procedures. Pires de Morais et al. emphasize that precise assessment of its dimensions—particularly the minimum diameter—is essential for appropriate valve selection and for minimizing the risk of complications such as paravalvular leakage [52]. Moreover, indexing the aortic valve calcification score to the aortic annulus area enables a more accurate evaluation of aortic stenosis severity and may reduce diagnostic uncertainty. A similar standpoint was presented by Cote et al., who normalized the amount of calcification to the cross-sectional area of the aortic annulus and demonstrated that this indexed measure correlates more strongly with the transvalvular pressure gradient than the CT-AVC score alone [53]. Additionally, Mousavi et al. highlight that in patients with small aortic valves, a lower calcium burden is required to induce marked stiffening and clinically significant stenosis [54].

In our analysis of potential predictors of aortic stenosis severity obtainable from cardiac computed tomography, we considered the aortic valve calcium score, calcium score indexed by aortic annulus area, aortic annulus area, aortic strain, aortic contractility, left ventricular ejection fraction, and left atrial volume. Some researchers emphasize that the severity of stenosis depends on the type of BAV. As noted by Fernandes et al., R-N valves are more likely to cause moderate and severe stenosis and significant regurgitation than R-L valves [55]. However, these studies were mostly based on the Sivers–Schmidtke classification and did not take into account the divisions proposed by Jilaihawi et al. and Michelena et al. Therefore, in the group of patients with BAV, we additionally included the morphological type of the valve based on the Jilaihawi classification.

Differentiating between a bicuspid and a tricuspid aortic valve is challenging in patients with calcific aortic stenosis, but as Masri et al. indicated, it remains clinically important for treatment decision-making [56]. Transthoracic echocardiography (TTE) is frequently associated with difficulties in obtaining an adequate acoustic window for visualizing a bicuspid aortic valve [57]. Uhlig et al. in doctoral dissertation compared the effectiveness of cardiac computed tomography and transthoracic echocardiography in detecting bicuspid aortic valves [58]. In all cases, intraoperative findings confirmed the valve type determined by ECG-gated CT. In echocardiographic reports, BAV was suspected in 76 individuals, whereas CT confirmed BAV in 61 of them (approximately 80%). Among 793 individuals in whom echocardiography did not suggest BAV, CT identified this defect in 30 cases (3.8%).

In echocardiography, AVA calculation is most commonly based on three measurements: LVOT diameter, aortic valve TVI (time–velocity integral), and LVOT TVI. As stated by Messika-Zeitoun et al. [14], each of these measurements is subject to error, which can substantially affect the final AVA value. For this reason, when assessing the relationship between AVA_CT_ and AVA_echo_, we included only echocardiographic examinations performed at our center in order to minimize inter-operator variability. Consequently, AVA_echo_ was included in the analysis for approximately one-third of patients in the TAV and BAV groups evaluated prior to planned TAVI. Some operators at our center assessed AVA using the planimetric method, whereas others used the continuity equation, which impacted the homogeneity of measurements. The choice of method depended on image quality and patient anatomy. When a precise delineation of the valve orifice was possible, planimetry was applied; however, when valve plane visualization was limited or haemodynamic considerations dominated, the continuity equation was preferred. As stated by Rong et al. in their meta-analysis, planimetric techniques tend to overestimate AVA compared to haemodynamic techniques [59]. Echocardiographic planimetric assessment may be difficult due to the limited resolution of the examination, especially in patients with severe calcification, which often characterizes patients with significant aortic stenosis. On the other hand, the continuity equation method is dependent on haemodynamic parameters and sensitive to measurement errors, particularly LVOT diameter [14,59,60]. Tops et al. emphasize that multislice computed tomography can guarantee accurate and non-invasive imaging not only of the coronary arteries, but also of the planimetry and geometry of the aortic valve, including the aortic annulus and LVOT [61]. Utsunomiya et al. point out that the shape of the LVOT in a significant proportion of patients is elliptical, which leads to underestimation of AVA measurements using the continuity equation (CE) in echocardiography [62]. In our study, AVA_CT_ correlated significantly with AVA_echo_ in the TAV group (R = 0.36, *p* = 0.039) but did not reach statistical significance in the BAV group (*p* = 0.180). It is important to note that this assessment was conducted in approximately one-third of the cohort, as we sought to improve group homogeneity by excluding echocardiographic examinations performed at external units (minimizing inter-operator variability). Moreover, in a substantial proportion of patients, the primary echocardiographic parameters assessed before TAVI were the maximum and mean transvalvular pressure gradients, obtained in approximately 60–70% of patients in the TAV and BAV groups. Seoudy et al. emphasize that planimetric assessment in echocardiography carries an inherent degree of inaccuracy, as extensive valve calcification produces acoustic shadowing and artefacts that may hinder reliable determination of AVA_echo_. Furthermore, in many cases (particularly in bicuspid aortic valves) accurate identification of the minimum orifice area is challenging [60]. This may be related to the greater calcification burden observed in the BAV group. In our study, the BAV and TAV groups were matched for age and sex, and differences in valve calcium score were close to significance (BAV: 4194.8 ± 2748.7 vs. TAV: 3335.0 ± 1618.8, *p* = 0.08). When analyzing men only, the difference became statistically significant (BAV: 5596.8 ± 2936.6 vs. TAV: 4061.4 ± 1659.8, *p* = 0.002).

Halpern et al. clearly demonstrated that there are differences between AVA_echo_ measured using the CE method and AVA_CT_ determined using the planimetric method. These differences proved to be statistically significant (*p* = 0.0037), with an average measurement discrepancy of 0.6 cm^2^. According to the authors, these divergences may be partly due to the different methods of LVOT assessment—based on LVOT diameter in the case of CE and direct planimetry of the LVOT area in CT [63]. In our study, we also noted significant discrepancies in the aortic valve area both in the BAV group (AVA_CT_ 167.5 ± 53.3 mm^2^ vs. AVA_echo_ 85.1 ± 29.8 mm^2^) and in the TAV group (AVA_CT_ 153.2 ± 41.2 mm^2^ vs. AVA_echo_ 83.0 ± 25.6 mm^2^). It is worth noting that in our study we analyzed only patients with clinically significant aortic stenosis qualified for TAVI, which may explain the differences compared to the results reported by Halpern et al.

Transvalvular aortic pressure gradients appear to be less susceptible to measurement errors than AVA, particularly in older patients with advanced valvular calcification [64]. In our study, we obtained satisfactory and statistically significant negative correlations between AVA_CT_ and both the mean and maximum transvalvular aortic pressure gradients measured by echocardiography: R = −0.43, *p* = 0.001 and R = −0.50, *p* < 0.001 for TAV, and R = −0.27, *p* = 0.038 and R = −0.36, *p* = 0.005 for BAV, respectively. Nevertheless, in a substantial proportion of cases with discrepancies between AVA_echo_ values and transvalvular gradients, transthoracic echocardiography is unable to accurately determine the true severity of aortic stenosis and requires additional diagnostic tests, including multislice computed tomography with ECG gating [14].

The studies published to date have focused mainly on assessing the usefulness of the aortic valve calcification score in evaluating the severity of aortic stenosis [16,19,31,32,33]. The linear regression models we have developed represent the first such approach to assessing the usefulness of other additional predictive parameters that can be obtained from cardiac computed tomography for estimating the severity of aortic valve stenosis in a group of patients eligible for TAVI. Both in univariate regression models and as a component of multivariate models, the Agatston (valve) score best explained the variability of the dependent variable—the aortic valve area. However, in the BAV group, the calcium score demonstrated markedly lower predictive performance, with an R^2^ of 0.224 in the univariate model. As Ryan et al. noted, in BAV, when the cusps begin to degenerate, calcifications tend to accumulate along the line of fusion and at the base of the fused cusps [65], which may indirectly affect the ability of the valve calcium score to explain AVA_CT_ variability. As stated by Fernandes et al., differences in the severity of aortic stenosis may also be related to the morphology of the bicuspid aortic valve [55], which is why our analysis also included BAV subtypes according to the Jilaihawi classification (intended for patients prior to TAVI). Demographic variables were not included in the analysis, as the focus was placed on parameters obtainable from multislice computed tomography. Given the slightly weaker explanatory power of the Agatston valve score for AVA_CT_, we performed a multivariate analysis using backward stepwise regression, both with and without statistically significant interactions between the main variables. The model without interactions achieved an R^2^ of 0.280 and additionally incorporated aortic strain and aortic annulus area. As stated by Santarpia et al., patients with bicuspid aortic valve undergo left ventricular remodeling, which is largely explained by the reduced aortic and left ventricular deformation properties and increased stiffness of the ascending aorta [66]. In addition, individuals with BAV experience hemodynamic flow disturbances manifested by increased shear stress on the aortic wall [67]. Therefore, it seems reasonable to consider aortic strain as a complementary indicator to the aortic valve calcium score in assessing the severity of aortic stenosis in the BAV group. Our analysis indicates that in patients with clinically significant aortic stenosis and preserved ascending aortic contractility, we can expect less advanced AS than in the group of patients with reduced aortic contractility, with similar Agatston score values. The second parameter included in the multivariate model was aortic annulus area. It is worth mentioning that the aortic valve calcium score indexed by the aortic annulus area explained AVA_CT_ variability in a way comparable to the traditional aortic valve calcium score in the univariate regression model (R^2^ = 0.218 vs. R^2^ = 0.224).

Patients with bicuspid aortic valves (BAV) exhibit considerable variability in aortic annulus morphology and valve calcification patterns. In the BAV group, calcifications more frequently involve the non-coronary cusp, particularly at the free edge of the leaflet [68]. Therefore, incorporating the aortic annulus area when assessing aortic stenosis severity in BAV patients is reasonable. Alternatively, indexing the valvular calcium score to the aortic annulus area could be considered; however, its predictive superiority over the conventional calcium score would need to be validated, ideally in a larger patient population. It is well known that mechanical strain plays a major role in the development of aortic valve calcification. Some authors have demonstrated substantial differences in flow patterns and mechanical strain between tricuspid and bicuspid aortic valves [68]. Similar heterogeneity can also be observed among bicuspid valve phenotypes, which differ markedly in both morphological characteristics and mechanical strain. These differences may ultimately result in an asymmetric distribution of valve calcifications. This heterogeneity likely explains why aortic valve calcium scoring predicts the severity of aortic stenosis more accurately in tricuspid valves, which represent a morphologically more homogeneous population.

In the next step, we performed multiple regression analysis including interactions. The only statistically significant interaction retained in the final model was the Agatston score × tricommissural BAV. The final model demonstrated a fit of R^2^ = 0.324. The variable tricommissural BAV was also retained in the model in accordance with the hierarchical principle to enhance model stability. Morphologically, tricommissural BAV most closely resembles the classic tricuspid valve, which is generally associated with less severe valve calcification. This finding was supported by its significant interaction with the aortic valve calcium score (Agatston score). Clinically, the tricommissural BAV subtype may be associated with less severe aortic stenosis compared to other subtypes in the Jilaihawi classification, suggesting a potential protective effect of this morphology. Kaira et al. emphasize that tricommissural BAV is often acquired, arising from rheumatic or degenerative processes, and thus manifests later in life [69]. Both models presented meet the fundamental assumptions of classical regression and provide an important alternative to isolated assessment of the calcium score, substantially improving model fit (R^2^). While the interaction model better explains AVA_CT_ variability, it also carries a higher risk of overfitting.

We built models in a similar manner for the group with tricuspid aortic valves. In this group, the Agatston score exhibited a much higher R^2^ in univariate regression analysis, reaching 0.479. Multiple regression using the stepwise backward method identified an additional statistically significant predictive factor—aortic contractility—which slightly increased the model’s R^2^ to 0.495. Singh et al. emphasize that aortic stenosis contributes to increased aortic stiffness [70]. Nevertheless, the effect of this variable on explaining AVA_CT_ variability appears modest, and in clinical practice, reliance on the isolated Agatston score seems more reasonable. The final model incorporating interactions included statistically significant interactions, such as left atrial volume × ejection fraction (*p* = 0.023) and aortic annulus area × ejection fraction (*p* = 0.012), alongside several main variables. All variables and interactions in the final model were centered to reduce collinearity and improve estimation precision, resulting in a final R^2^ of 0.576. Despite the improved fit, this model was less transparent and carried a higher risk of overfitting, limiting its practical clinical applicability.

In our study, we considered potential predictive parameters obtainable from cardiac computed tomography but did not include demographic factors. Nevertheless, the groups of patients with bicuspid and tricuspid aortic valves were matched for age and gender. Demographic variables (including age) were excluded from the models because the study population consisted of patients eligible for TAVI, representing a cohort that was inherently homogeneous in terms of age (predominantly elderly). Consequently, age variation was slight and provided little predictive information. Constructing separate models stratified by gender would have further subdivided the sample, increasing the risk of overfitting and reducing model reliability. Therefore, for both methodological and statistical reasons, demographic variables were not included in the final models. Due to the limited sample size, the entire available set of variables was used to build the model. It was not possible to create an independent test set; thus, model quality was assessed through internal analysis (cross-validation) and evaluation of fit stability.

The complex regression models we propose, incorporating interactions, are primarily exploratory and theoretical in nature. Models with interaction terms are inherently more flexible and, therefore, more prone to capturing random variation within a single dataset. Prospective validation in future studies will allow for standardized, high-quality measurement of all interacting variables and outcomes. This approach minimizes the risk of recall or information bias, which can otherwise lead to spurious interaction effects in regression models. On the other hand, models based solely on main variables, which are characterized by significantly greater estimation stability, may serve as a practical tool for the preliminary assessment of aortic stenosis severity in patients eligible for TAVI. However, the predictive value of these models should be considered carefully, particularly in patients with bicuspid aortic valves, in whom anatomical heterogeneity may substantially affect the accuracy of predictions.

This study is limited by its retrospective design. Prospective studies conducted in larger patient populations are warranted to further evaluate the clinical utility of the predictive factors identified. Because the data were not collected using a predesigned proforma tailored to the specific aims of the study, some degree of missing data was unavoidable. In the present analysis, this limitation primarily reflected restricted access to perioperative clinical data, which precluded calculation of the overall incidence of TAVI-related complications in the entire study cohort, as well as incomplete echocardiographic measurements, most frequently involving aortic valve area. The lack of a significant correlation between CT-derived and echocardiographic aortic valve area in the bicuspid aortic valve subgroup may be partially explained by the fact that this analysis was performed in approximately one-third of the study cohort to ensure single-center data homogeneity. Moreover, in patients with bicuspid aortic valves, echocardiographic assessment of aortic valve area is technically more challenging than in tricuspid valves due to asymmetric valve geometry, eccentric flow jets, and the limited accuracy of two-dimensional planimetry and left ventricular outflow tract measurements, which may have further influenced this analysis.

## 5. Conclusions

Linear regression models determine the severity of aortic stenosis in patients scheduled for TAVI more accurately than any individual predictive parameter derived from ECG-gated cardiac CT.

The Agatston AVC score appears to be the most reliable single predictive parameter obtainable from cardiac computed tomography for assessing the severity of aortic stenosis in a group of patients with both tricuspid and bicuspid aortic valves prior to planned TAVI.

The predictive value of the valve calcium score in estimating the severity of aortic stenosis prior to TAVI is significantly lower in patients with BAV than in those with TAV; therefore, it may be reasonable to evaluate additional parameters such as the aortic strain and aortic annulus area.

The aortic valve area measured by cardiac computed tomography correlates well with aortic valve gradients obtained by echocardiography. Further comparative studies in larger patient cohorts, using a standardized echocardiographic method for measuring the aortic valve area, are needed to assess the relationship between the aortic valve area determined by computed tomography and that measured by echocardiography, particularly in patients with BAV.

Patients eligible for TAVI with a functional bicuspid aortic valve (tricommissural BAV according to Jilaihawi) appear to have less severe aortic stenosis compared to other BAV subtypes. However, this observation still needs to be confirmed in further prospective studies involving larger cohorts.

## Figures and Tables

**Table 1:** Characteristics of the study group. * In the analysis of the rate of TAVI-related complications, 70 patients in the BAV group and 75 patients in the TAV group were included, based on the availability of perioperative medical data.

	BAV (n = 105)	TAV (n = 105)
Age [years], Me (Min–Max)	77 (59–90)	77 (59–90)
Sex-Females, n (%)	57 (54.3%)	57 (54.3%)
AVA_CT_ [mm^2^], Mean ± SD	167.5 ± 53.3	153.2 ± 41.2
Agatston score (Ca) [AU], Me (Min-Max)	4032 (509–13,592)	2930 (529–8286)
Ca/AVAA index [AU/cm^2^], Me (Min–Max)	678.5 (76.5–1883.0)	619.0 (151.1–1466.5)
Aortic strain [%], Me (Min–Max)	2.7% (0.0–6.3%)	2.9% (1.6–6.5%)
Aortic contractility [%], Me (Min–Max)	5.3% (0.8–20.5%)	5.5% (1.6–18.3%)
Aortic annulus area [cm^2^], Me (Min–Max)	554 (282–1141)	498 (221–837)
Left atrial volume [mL], Me (Min–Max)	149 (67–700)	165 (70–387)
LV ejection fraction [%], Me (Min–Max)	63 (12–86)	66 (28–90)
**Complications related** to TAVI *		
Stroke, n (%)	2 (1.9%)	1 (0.9%)
Conduction disturbance, n (%)	14 (13.3%)	15 (14.3%)
Vascular complication, n (%)	10 (9.5%)	8 (7.6%)
Paravalvular regurgitation, n (%)	8 (7.6%)	5 (4.8%)
30-day mortality, n (%)	4 (3.8%)	3 (2.9%)
Coronary obstruction, n (%)	2 (1.9%)	1 (0.9%)
Annular rupture, n (%)	1 (0.9%)	0 (0%)

**Table 2:** Classification of patients with BAV according to the Sivers–Schmidtke classification.

Type	Type 0		Type 1		
Subtype	0 lateral	0 A-P	1 RL	1 NR	1 NL
N (%)	11 (10.5%)	0 (0.0%)	72 (68.6%)	16 (15.2%)	6 (5.7%)

**Table 3:** Classification of patients with BAV according to the Jilaihawi classification.

Tricommisural	Bicommissural Raphe-Type	Bicommissural Non-Raphe-Type
47 (44.8%)	47 (44.8%)	11 (10.5%)

**Table 4:** Assessment of the relationship between aortic valve area measured by cardiac computed tomography and the most important echocardiographic parameters (maximum aortic gradient, mean aortic gradient, aortic valve area) in the TAV and BAV groups. * n = 68, ** n = 33, *** n = 60, **** n = 30. AVA—aortic valve area. TAV—tricuspid aortic valve. BAV—bicuspid aortic valve.

	AVA_CT_, TAV		AVA_CT_, BAV	
Parameter	Mean ± SD	R, *p*	Mean ± SD	R, *p*
maximum aortic pressure gradient, [mm Hg]	77.9 ± 23.5 *	R = −0.50, *p* < 0.001	78.7 ± 24.7 ***	R = −0.36, *p* = 0.005
mean aortic pressure gradient, [mm Hg]	46.8 ± 15.8 *	R = −0.43, *p* = 0.001	48.0 ± 15.8 ***	R = −0.27, *p* = 0.038
AVA_echo_, [mm^2^]	83.0 ± 25.6 **	R = 0.36, *p* = 0.039	85.1 ± 29.8 ****	R = 0.25, *p* = 0.180

**Table 5:** Characteristics of potential predictive parameters of aortic stenosis in the TAV and BAV groups, presented as Mean ± SD.

Variable	TAV	BAV
Agatston score (Ca) [AU]	3335.0 ± 1618.8	4194.8 ± 2735.5
Ca/AVAA index [AU/cm^2^]	638.4 ± 256.5	707.7 ± 391.4
Aortic strain [%]	3.4 ± 1.2	3.2 ± 1.2
Aortic contractility [%]	6.7 ± 3.7	6.7 ± 3.7
Aortic annulus area [cm^2^]	514.3 ± 105.4	579.1 ± 138.2
Left atrial volume [ml]	167.2 ± 44.7	163.2 ± 72.1
LV ejection fraction [%]	64.1 ± 14.0	61.3 ± 15.3

**Table 6:** Results of simple linear regression analysis in the BAV and TAV groups.

Variable	BAV				TAV			
	β	95% CI	*p*	R^2^	β	95% CI	*p*	R^2^
Agatston score (Ca) [AU]	−0.009	(−0.013, −0.005)	**<0.001**	0.224	−0.018	(−0.023, −0.013)	**<0.001**	0.479
Ca/AVAA index [AU/cm^2^]	−0.064	(−0.087, −0.040)	**<0.001**	0.218	−0.068	(−0.085, −0.050)	**<0.001**	0.389
Aortic strain [%]	6.665	(−1.730, 15.060)	0.121	0.023	−5.074	(−11.826, 1.679)	0.138	0.021
Aortic contractility [%]	1.819	(−0.950, 4.590)	0.201	0.016	−1.052	(−3.197, 1.094)	0.340	0.009
Aortic annulus area [cm^2^]	−0.050	(−0.125, 0.025)	0.188	0.017	−0.058	(−0.109, −0.007)	**0.026**	0.041
Left atrial volume [mL]	−0.105	(−0.248, 0.038)	0.150	0.020	−0.253	(−0.425, −0.082)	**0.005**	0.075
LV ejection fraction [%]	0.103	(−0.574, 0.780)	0.767	0.001	0.079	(−0.472, 0.630)	0.793	0.001
Type: Bicommissural Raphe-type vs. bicommissural Non Raphe-type	13.360	(−7.244, 33.964)	0.201	0.016				
Type: Tricommissural vs. bicommissural Non Raphe-type	−5.048	(−25.907, 15.812)	0.632	0.002				

**Table 7:** Components of the multiple regression model in the TAV group.

Variable	B	*p*-Value
const	229.650	<0.001
Agatston score	−0.020	<0.001
Aortic annulus area	0.097	0.009
Aortic contractility	−165.260	0.043
Left atrial volume	−0.170	0.024

**Table 8:** Components of the multiple regression model in the TAV group, after excluding the variables—left atrial volume and aortic annulus area.

Variable	B	*p*-Value
const	229.650	<0.001
Agatston score	−0.018	<0.001
Aortic contractility	−142.205	0.050

**Table 9:** Components of the multiple regression model in the TAV group, taking into account interactions between variables. AAA—aortic annulus area, LAV—left atrial volume, EF—ejection fraction.

Variable	B	*p*-Value
const	229.650	<0.001
Agatston score_c_	−0.020	<0.001
Aortic annulus area_c_ (AAA)	0.089	0.015
Aortic contractility_c_	−212.983	0.009
Left atrial volume_c_ (LAV)	−0.170	0.024
Ejection fraction_c_ (EF)	−0.433	0.067
AAA × EF	−0.006	0.012
LAV × EF	0.011	0.023

**Table 10:** Components of the multiple regression model in the BAV group.

Variable	B	*p*-Value
const	165.963	<0.001
Agatston score	−0.011	<0.001
aortic strain	795.900	0.037
aortic annulus area	0.077	0.052

**Table 11:** Components of the multiple regression model in the BAV group, taking into account interactions between variables.

Variable	B	*p*-Value
const	146.849	<0.001
Agatston score	−0.008	<0.001
aortic strain	874.633	0.021
aortic annulus area	0.088	0.027
tricommissural BAV	26.281	0.119
Agatston score × tricommissural BAV	−0.008	0.019

## Data Availability

The original contributions presented in the study are included in the article, further inquiries can be directed to the corresponding author.
